# A Brief Review of Demographic and Clinical Correlates of Cholesteatoma Surgery in the Qassim Region

**DOI:** 10.7759/cureus.35676

**Published:** 2023-03-02

**Authors:** Waleed A Alhazmi, Mohammed H Al Mansour, Renad I Aljasser, Afaf M Alanazi, Saja D Alyami, Abdulaziz B Almutairi, Ibrahim N Al Sulaiman

**Affiliations:** 1 Otorhinolaryngology, Qassim University, Buraydah, SAU; 2 Otorhinolaryngology, Najran General Hospital, Najran, SAU; 3 Medicine, Qassim University, Buraidah, SAU; 4 Intensive Care Unit, Armed Forces Hospitals Southern Region, Khamis Mushait, SAU; 5 Medicine, Majmaah University, Majmaah, SAU; 6 Medicine, Najran University, Najran, SAU

**Keywords:** middle ear disease, clinical correlates, demographics, comorbidities, cholesteatoma

## Abstract

Background

Cholesteatoma is described as the accumulation of squamous epithelium and keratinocytes within and around the middle ear cleft. There is a paucity of information regarding demographic and treatment outcomes for cholesteatoma in Saudi Arabia. An evaluation of the prevalence of comorbidities, complications and associations, of surgical treatment and demographics in the Qassim region was conducted.

Methods

This was a six-year retrospective review of patients treated for cholesteatoma at a private health facility, from August 2016 to July 2022. Data for age, gender, nationality, presence of comorbidities, type of surgery, type of anesthesia, and associated complications were collected from the electronic medical records and analyzed with Statistical Package for Social Sciences software.

Results

A total of 60 participants records were retrieved. The average age of the study population was ([43.2 ±SD] 21.8) years. There was a slightly higher male preponderance (males 51.7% and females 48.3%). Hypertension was the most commonly reported comorbidity (31.7%), followed by diabetes mellitus (25%). Age and gender were not statistically significantly associated with type of surgery or complications.

Conclusion

Demographic variables were not significantly associated with clinical correlates, however, further studies with larger sample sizes, robust clinical information, and long-term follow-up are required.

## Introduction

Cholesteatoma is a non-neoplastic accumulation of keratinizing stratified squamous epithelium trapped within the middle ear space that can erode and cause bone erosion [1]. Bone erosion can lead to more serious complications by the spread of infection outside the middle ear and this infection can form a bony abscess, labyrinthitis, facial nerve paralysis, and spread to the brain and cause meningitis or a brain abscess [1]. An untreated middle ear cholesteatoma can lead to serious complications such as hearing loss and deafness [2,3].

Currently, there are several surgical techniques for the treatment of cholesteatoma, each with its merits and demerits. The only surgical procedure to remove cholesteatomas in the early 1900s was radical mastoidectomy [4]. Tympanoplasty had gained widespread acceptance by the 1950s, and the combined tympanomastoidectomy operation is still performed nowadays [5]. The “canal-wall-down” (CWD) and “canal-wall-up” (CWU) are the hallmark techniques of tympanomastoidectomy for cholesteatoma management. Simply put, the main difference is the destruction or preservation of the posterior canal wall respectively. However, the decision for any one surgical approach rests largely on the surgeon’s skill, discretion, and preferences.

Cholesteatomas affect all age groups and can be classified as one of two types: congenital, which is specific to childhood, and acquired, which affects children as well as adults [6]. The congenital type of cholesteatoma is a white mass that forms prior to birth behind an intact eardrum, with no associated history of otitis media or previous otologic procedures [7]. While acquired cholesteatoma usually begins after birth with a retraction pocket on the eardrum, usually following a chronic middle ear infection [7].

The annual incidence of acquired cholesteatoma ranges from approximately nine to 13 cases per 100,000 adults and three to 15 cases per 100,000 children [8,9]. In recent decades, the incidence of acquired cholesteatoma has declined due to the widespread use of ventilation tubes [10]. Compared to adult cholesteatomas, pediatric cholesteatomas are more often infectious, more aggressive, and hyperproliferative, as well as having a worse prognosis [11].

The prevalence of cholesteatoma is said to be higher in underdeveloped countries than in developed countries [8] and among the socially deprived [12]. Children of lower-income families are more prone to complications such as subperiosteal abscesses and acute mastoiditis in comparison to those of higher-income families [13].

Saudi Arabia is a middle-high-income country, and its economy is one of the best in the region, as such medical and disease prevention services have also improved; as a result, it has developed an increasingly “aging society.” Therefore, an evaluation of trends in otologic surgeries according to morbidities and sociodemographics is crucial for national health planning.

To our knowledge, there are no reports on cholesteatoma treatment outcomes in the region. The aim of this study was to evaluate the prevalence of comorbidities, complications, and associations, if any, of surgical technique and demographics in a private health facility in the Qassim region, of Saudi Arabia.

## Materials and methods

This was a six-year retrospective review of medical records of patients admitted with a diagnosis of cholesteatoma at a private health facility, Dr. Sulaiman Al Habib Hospital, in the Qassim region of Saudi Arabia, from August 2016 to July 2022.

A total of 60 patients' medical records were retrieved for the study. Clinical information such as age, gender, nationality, presence of comorbidities, type of surgery, type of anesthesia, and any associated complications was obtained for review and analysis.

Data analysis

The collected data was initially entered into a Microsoft Excel file (Microsoft Corp., New Mexico, USA) and then transferred to SPSS (for windows, SPSS, IBM Corp., Chicago, USA, version 25) software. Quantitative data were summarized using frequencies and percentages, while qualitative data were analyzed with Chi-square crosstabs to determine the relationship between variables, with a value of P < 0.05 used for evaluating statistical significance (95% Confidence interval).

Ethical clearance

Permission and ethical approval were obtained from the Institutional Review Board Committee, The Research Center at Dr. Sulaiman Al Habib Medical Group, Riyadh, Saudi Arabia, with study number RC22.10.19. This study was conducted according to the ethical principles of the World Medical Assembly revised declaration of Helsinki (2013).

## Results

A total of 60 patients were included in the study. The majority were Saudis (93.3%) while the average age of the patients was 43.2 ± 21.8 years (range 9-93), with a slightly higher male preponderance (males 51.7% and females 48.3%) (Table 1). While investigating the associated comorbidities of the patients, hypertension was found to be the most common documented comorbidity (31.7%). Other comorbidities are shown in Table 1. Further analysis of the records shows that general anesthesia was the singular mode of anesthesia used for cholesteatoma extirpation. According to the records, no complications were recorded during the period under review.

**Table 1:** Demographic and clinical characteristics of the study participants (n=60)

Variable		Mean ± SD	Range
Age (in years)		43.2 ± 21.8	9-93
Variable	Category	Frequency	Percent
Nationality	Saudi	56	93.3
	Non-Saudi	4	6.7
Gender	Male	31	51.7
	Female	29	48.3
Comorbidity	Diabetes mellitus	15	25
	Hypertension	19	31.7
	Hyperlipidemia	3	5
	Rheumatoid arthritis	1	1.7
	Deviated nasal septum	13	21.7
	Down syndrome	1	1.7
	Asthma	1	1.7
	Allergic rhinitis	14	23.4
	Adenoid hypertrophy	6	10
Type of anesthesia	Local	0	0
	General	60	100
Complications	Yes	0	0
	No	60	100

Type of procedure and its association with gender and age of the participants

Our results indicated that tympanomastoidectomy was the most frequently documented procedure conducted for both genders; males 45.2% and females 34.5%. This was followed by tympanoplasty, which was conducted for 25% of all patients. Other procedures were listed in Table 2. In addition, we found that gender and age did not show any significant association with the type of procedure (Tables 2, 3).

**Table 2:** Type of procedure and its association with the gender of the participants * P-value calculated using chi-square test, other p values calculated using Fisher's exact test.

Variable	Overall N (%)	Gender		P-value
		Male N (%)	Female N (%)	
Tympanomastoidectomy	24 (40)	14 (45.2)	10 (34.5)	0.399*
Tympanoplasty	15 (25)	9 (29)	6 (20.7)	0.456*
Radical mastoidectomy	4 (6.7)	1 (3.2)	3 (10.3)	0.346
Modified Radical mastoidectomy	2 (3.3)	0 (0)	2 (6.9)	0.229
Simple Mastoidectomy	2 (3.3)	2 (6.5)	0 (0)	0.492
Atticotomy with tympanoplasty	2 (3.3)	1 (3.2)	1 (3.4)	1.000
Atticotomy	1 (1.7)	0 (0)	1 (3.4)	0.483
Cortical mastoidectomy	1 (1.7)	0 (0)	1 (3.4)	0.483
Cortical mastoidectomy , tympanoplasty, and ossiculoplasty	1 (1.7)	0 (0)	1 (3.4)	0.483
Modified radical mastoidectomy with tympanoplasty	1 (1.7)	0 (0)	1 (3.4)	0.483
Radical mastoidectomy with tympanoplasty	1 (1.7)	1 (3.2)	0 (0)	1.000
Simple mastoidectomy, tympanoplasty, and ossiculoplasty	1 (1.7)	1 (3.2)	0 (0)	1.000
Atticotomy , tympanoplasty, and canaloplasty with meatoplasty	1 (1.7)	0 (0)	1 (3.4)	0.483
Ossiculoplasty	1 (1.7)	1 (3.2)	0 (0)	1.000
Turbinectomy	1 (1.7)	0 (0)	1 (3.4)	0.483
Adenoidectomy	1 (1.7)	0 (0)	1 (3.4)	0.483
Adenoidectomy with bilateral myringotomy and tubes	1 (1.7)	1 (3.2)	0 (0)	1.000

**Table 3:** Type of procedure and its association with the age of the participants * P-value calculated using independent samples t-test.

Variable		Age (in years)		P-value
		Mean	SD	
Tympanomastoidectomy	Yes	45.83	23.94	0.444
	No	41.39	20.41	
Tympanoplasty	Yes	38.60	23.12	0.353
	No	44.69	21.39	
Radical mastoidectomy	Yes	54.50	11.00	0.286
	No	42.36	22.21	
Modified Radical mastoidectomy	Yes	61.50	2.12	0.229
	No	42.53	21.90	
Simple Mastoidectomy	Yes	44.50	16.26	0.931
	No	43.12	22.07	
Atticotomy with tympanoplasty	Yes	35.50	7.78	0.617
	No	43.43	22.11	
Atticotomy	Yes	50.00	-	0.755
	No	43.05	21.96	
Cortical mastoidectomy	Yes	11.00	-	0.138
	No	43.71	21.57	
Cortical mastoidectomy, tympanoplasty, and ossiculoplasty	Yes	59.00	-	0.469
	No	42.90	21.89	
Modified radical mastoidectomy with tympanoplasty	Yes	50.00	-	0.755
	No	43.05	21.96	
Radical mastoidectomy with tympanoplasty	Yes	22.00	-	0.332
	No	43.53	21.81	
Simple mastoidectomy, tympanoplasty, and ossiculoplasty	Yes	33.00	-	0.642
	No	43.34	21.95	
Atticotomy , tympanoplasty, and canaloplasty with meatoplasty	Yes	70.00	-	0.217
	No	42.71	21.70	
Ossiculoplasty	Yes	59.00	-	0.469
	No	42.90	21.89	
Turbinectomy	Yes	30.00	-	0.547
	No	43.39	21.92	
Adenoidectomy	Yes	13.00	-	0.165
	No	43.68	21.62	
Adenoidectomy with bilateral myringotomy and tubes	Yes	13.00	-	0.165
	No	43.68	21.62	

## Discussion

This study revealed a higher prevalence of cholesteatoma among Saudi adult males than non-Saudis and females and, consequently, had more surgeries for cholesteatoma via general anesthesia. Hypertension was the commonest comorbidity documented; this is not surprising, especially among male patients. Hypertension is said to be increasing in prevalence in Saudi Arabia affecting more than one-fourth of the adult Saudi population [14] and about 58% of hypertensive Saudis are undiagnosed, were more likely to be male, older, as well as having a diagnosis history of diabetes mellitus [15]. 

Regarding the age of occurrence, cholesteatomas are thought to be more common in adults, but they tend to behave more aggressively and carry a worse prognosis in children [13]. Although our review did not include children but concurs with the high prevalence among adults. The study also highlights the higher propensity of cholesteatomas among males and is corroborated by many previous studies as well [5,9].

The safety of general anesthesia has substantially improved over the last two decades, particularly with regard to hypotensive anesthesia, resulting in a significant decrease in the number of patients considered medically incompetent for general anesthesia [12]. In our review, general anesthesia was the anesthetic of choice for the extirpation of cholesteatoma for all the patients as well.

In terms of intra or postoperative complications, we did not find any documented complications for the patient population. Whereas many studies document complications from the records, e.g., a study conducted in Saudi Arabia found that 105 patients (10.2%) experienced postoperative problems of which minor complications were the most common (9.5%) [16], and many other studies elsewhere [12,16,17]. In relation to gender and age, one study [18] reported that the female gender has a protective effect against intraoperative complications as well as patient age which was found to be correlated with a higher incidence of intraoperative complications [18]. In our humble opinion, this omission may be attributable to reporting bias.

A report from Korea in 2018 shows that the male population had 1.2 times higher rates of cholesteatoma surgery in comparison to the female population [19]. According to this report, cholesteatoma surgery was most commonly performed in patients in their 50s. This was our observation as well with the slight male preponderance and near age bracket. Similarly, from our audit, we could not find any relationship or statistical significance between the type of operation and age and gender.

Many cholesteatomas and tympanomastoid surgery classifications have been established in order to describe the severity of the cholesteatoma and the risk of recurrence, as well as to compare surgical outcomes and surgical methods [20,21]. A noteworthy effort was recently made to develop a standard language for middle ear and temporal bone surgery for cholesteatoma [22,23]. Although the ideal strategy for cholesteatoma surgery is still being debated, the primary goal of surgery should be to produce a dry ear free of illness. However, given the changing clinical profile of patients presenting with cholesteatoma, it is critical to begin managing cholesteatoma not only from the standpoint of a safe and dry ear but also from the standpoint of a functionally improved ear. To achieve the greatest results, surgical care of cholesteatoma requires a three-dimensional strategy [24]. Treatment of severe and recurring cholesteatoma necessitates a highly personalized strategy that considers anatomic, clinical, and social aspects to determine the most successful surgical treatment choice [25].

This study has some limitations. It is a retrospective hospital-based study, with a small sample size thereby affecting reliable effect size estimations. Moreover, complete socioeconomic variables were not documented, and as such reliable discussions about the social determinants of cholesteatoma surgery as well as population-based conclusions cannot be made.

## Conclusions

The results of this study show that perhaps hypertension should be actively addressed in the management of patients with cholesteatoma. Demographic variables were not significantly associated with clinical correlates; however, further studies with larger sample sizes, robust clinical information, and long-term follow-up are needed to make more reliable clinical recommendations.
